# OP3‐4 peptide sustained‐release hydrogel inhibits osteoclast formation and promotes vascularization to promote bone regeneration in a rat femoral defect model

**DOI:** 10.1002/btm2.10414

**Published:** 2022-10-11

**Authors:** Peng Luo, Jiarui Fang, Dazhi Yang, Lan Yu, Houqing Chen, Changging Jiang, Rui Guo, Tao Zhu, Shuo Tang

**Affiliations:** ^1^ Department of Sport Medicine Huazhong University of Science and Technology Union Shenzhen Hospital (Nanshan Hospital) Shenzhen China; ^2^ Department of Spine Surgery Huazhong University of Science and Technology Union Shenzhen Hospital (Nanshan Hospital) Shenzhen China; ^3^ Department of Laboratory Medicine Huazhong University of Science and Technology Union Shenzhen Hospital (Nanshan Hospital) Shenzhen China; ^4^ Key Laboratory of Biomaterials of Guangdong Higher Education Institutes, Department of Biomedical Engineering Jinan University Guangzhou China; ^5^ Department of Respiratory and Critical Care Medicine, and Preclinical Research Center Suining Central Hospital Sichuan China; ^6^ Department of Orthopaedics, The Eighth Affiliated Hospital Sun Yat‐sen University Shenzhen China

**Keywords:** anti‐CXCL9 antibody, femur defect, OP3‐4 peptide, osteogenic differentiation, vascularization

## Abstract

Bone injury caused changes to surrounding tissues, leading to a large number of osteoclasts appeared to clear the damaged bone tissue before bone regeneration. However, overactive osteoclasts will inhibit bone formation. In this study, we prepared methacrylylated gelatin (GelMA)‐based hydrogel to co‐crosslink with OP3‐4 peptide, a receptor activator of NF‐κB ligand (RANKL) binding agent, to achieve the slow release of OP3‐4 peptide to inhibit the activation of osteoclasts, thus preventing the long‐term existence of osteoclasts from affecting bone regeneration, and promoting osteogenic differentiation. Moreover, CXCL9 secreted by osteoblasts will bind to endogenous VEGF and inhibit vascularization, finally hinder bone formation. Thus, anti‐CXCL9 antibodies (A‐CXCL9) were also loaded in the hydrogel to neutralize excess CXCL9. The hydrogel slow released of OP3‐4 cyclic peptide and A‐CXCL9 to simultaneously inhibiting osteoclast activation and promoting vascularization, thereby accelerating the healing of femur defect. Further analysis of osteogenic protein expression and signal pathways showed that the hydrogel may be through activating the AKT‐RUNX2‐ALP pathway and ultimately promote osteogenic differentiation. This dual‐acting hydrogel can effectively prevent nonunion caused by low vascularization and provide long‐term support for the treatment of bone injury.

## 1 INTRODUCTION

Femur fractures and defects caused by surgical resection of osteosarcoma are usually accompanied by two major problems in clinical treatment: nonunion of the fracture^1^ and avascular necrosis of the femur head.^2^, ^3^ The incidence of femur fractures is concentrated in the elderly, and the treatment of osteosarcoma usually requires the removal of a large amount of cancerous bone tissue, both of two situations are often difficult to treat through autologous bone transplantation.^3^ By designing a new type of injectable in situ forming hydrogel to promote bone formation and vascularization, it is expected to replace autologous bone transplantation and become a new treatment method for the treatment of femur injuries.^4^, ^5^, ^6^

As a three‐dimensional structure network with high water content, hydrogel is beneficial to the delivery of nutrients and the growth of blood vessels.^7^, ^8^ In addition, the hydrogel can also be used as a drug carrier to achieve sustained release of drugs or polypeptides.^9^, ^10^, ^11^ OP3‐4 polypeptide is a nuclear factor‐κB receptor activator ligand (RANKL) binding peptide, which inhibits the formation of osteoclasts by inhibiting the binding of RANKL to RANK on the surface of osteoclasts.^12^, ^13^, ^14^ Recent studies have also shown that OP3‐4 polypeptide binds to RANK on the surface of osteoblasts, induces membrane receptor aggregation, thereby enhancing osteoblast differentiation.^12^, ^14^, ^15^ Therefore, OP3‐4 can simultaneously inhibit osteoclast activation and promote osteogenic differentiation during bone tissue regeneration. The slow release of OP3‐4 polypeptide will help bone tissue regeneration.

However, in the process of bone tissue regeneration, another problem faced is the vascularization of bone tissue. Insufficient vascularization of bone tissue during the repair process can lead to problems such as bone nonunion.^1^ Nevertheless, it is worth noting that the osteoblasts can secrete Chemokine (C‐X‐C motif) ligand 9 (CXCL9), and CXCL9 will binds to endogenous vascular endothelial growth factor (VEGF) to prevent blood vessel formation.^16^, ^17^ Therefore, coordinating osteoblast differentiation and vascularization processes can further accelerate bone tissue healing. The use of anti‐CXCL9 antibodies (A‐CXCL9) was able to bind excess CXCL9, thereby directly abolishing the effect of CXCL9 on VEGF and improving the vascularization process.^16^

Realizing the long‐term sustained release of the drug or bioactive factor is the key to ensuring that the implant can play a role in promoting bone formation for a long time. After the polypeptide has been modified by methacrylation, it can be co‐cross‐linked with the methacryloyl polymer material to fix the polypeptide in the hydrogel to achieve long‐term sustained release of the polypeptide.^9^ In addition, methacrylated gelatin (GelMA) cannot only combine with polypeptides to achieve sustained release but also promote cell adhesion, proliferation.^18^ It also has adjustable mechanical properties to adapt to different tissue applications.^19^, ^20^ PCL‐PEG‐PCL (PCEC) copolymer is a nano micelle composed of medical polymers PCL and PEG. The raw material has extremely high biosafety and PCEC can be used to load drugs or growth factors to achieve long‐term sustained release.^21^, ^22^

In order to promote bone regeneration while reducing the effect of CXCL9 secreted by osteoblasts on the vascularization process, we designed a GelMA‐based hydrogel that sustained‐release osteogenesis‐promoting polypeptide OP3‐4 and anti‐CXCL9 antibody embedded in PCEC nanoparticles. In this study, we verified the sustained‐release effect of the hydrogel on A‐CXCL9, and the inhibitory effect on osteoclasts and the pro‐differentiation of rBMSCs into osteoblasts from the biofunctional aspect. Finally, the repair effect of slow‐release OP3‐4 and A‐CXCL9 hydrogel materials on femoral defects was evaluated in a rat femoral defect model, which proved that promoting vascularization can better assist bone regeneration.

## 2 MATERIALS AND METHODS

### 2.1 Materials

Gelatin, from cold‐water fish skin, was purchased from Sigma‐Aldrich (Shanghai, China). Methacrylic anhydride, ε‐caprolactone and polyethylene glycol (PEG) (Mw = 4000) were purchased from Macklin (Shanghai, China). Tin (II) 2‐ethylhexanoate and sodium laurylsulfonate were purchased from Aladdin (Shanghai, China). Ethylacetate, acetone, petroleum ether and dichloromethane were purchased from Sinopharm Chemical Reagent Co., Ltd (Shanghai, China). Lithium phenyl(2,4,6‐trimethylbenzoyl) phosphinate (LAP) was purchased from Shanghai Yinchang New Material Biological Co., Ltd. Methylamidated OP3 peptide (YCEIEFCYLIR) was purchased from Jiangsu Ji Tai Peptide Industry Science and Technology Co., Ltd. Streptomycin sulfate, penicillin, fetal bovine serum and trypsin were purchased from Gibco (Shanghai, China). Cell counting kit 8 was purchased from Bioss antibodies (Beijing, China). Live/Dead staining kit was purchased from KeyGEN BioTECH (Jiangsu, China). TRITC Phalloidin was purchased from YEASEN (Shanghai, China). Rabbit Anti‐RUNX2 antibody, Alizarin Red S Staining Kit, DAPI staining, and Anti‐Osteocalcin Rabbit pAb were purchased from Servicebio (Wuhan, China). Rat bone marrow mesenchymal stem cells were purchased from ChuangSeed Biomaterials.

### 2.2 Synthesis of methacrylylated gelatin

GelMA was synthesized as previously described.^23^ Briefly, the solution of gelatin was prepared at a concentration of 8% in a water bath at 60°C. Then, methacrylic anhydride was added dropwise to the gelatin solution at a ratio of 0.6:1 (methacrylic anhydride to gelatin). The reaction was kept under room temperature for 8 h. Finally, the solution was dialyzed against deionized water with cellulose dialysis bag (MwCO = 3500) for 5 days and centrifuged at 8000 rpm for 5 min to remove undissolved impurities. The supernatant after centrifuge was lyophilized at −80°C to obtain the final product GelMA.

### 2.3 Synthesis of PCEC and A‐CXCL9@PCEC nanoparticles

PCEC nanoparticles were obtained following the previous report.^21^ A 4 g of PEG and 96 g of anhydrous ε‐caprolactone were added to dry three‐necked bottle and few drops of tin (II) 2‐ethylhexanoate was added to the above solution. The mixture was kept at 130°C for 6 h. Subsequently, the air in the reaction device was exhausted, and the mixture was heated to 180°C under vacuum and kept for 30 min. The mixture was then cooled to room temperature under the protection of nitrogen and dissolved in dichloromethane, and then the product PCEC was precipitated with excess cold petroleum ether, and finally filtered and dried to obtain a PCEC copolymer.

The anionic PCEC nanoparticles were then derived by the following methods^24^: 30 mg of PCEC copolymer was dissolved in 5 ml of acetone/ethyl acetate, and then 5 mg of sodium laurylsulfonate was dissolved in 10 ml of water and added to PCEC solution. Stir at 10,000 rpm to form a sodium dodecyl sulfonate emulsion and finally remove most of the solvent by rotary evaporation under reduced pressure. The resulting slurry was dialyzed to remove sodium dodecyl sulfonate. PCEC and A‐CXCL9 were mixed and stirred at 4°C for 24 h to obtain the final product A‐CXCL9@PCEC nanoparticles.

The amount of A‐CXCL9 was detected by ELISA kit. The ELISA kit was obtained by double antibody sandwich method. Anti‐CXCL9 antibody was purchased from Abcam (Shanghai, China). Recombinant Murine MIG (CXCL9) was purchased from Peprotech (Shanghai, China). Biotin‐NHS was purchased from Sigma‐Aldrich. HRP‐labeled Streptavidin was purchased from Sangon Biotech (Shanghai, China).

### 2.4 Preparation of the hydrogel

GelMA, OP3‐MA, and A‐CXCL9@PCEC were dissolved in PBS buffer according to a certain concentration ratio. LAP was then dissolved in the above mixture at the final concentration of 0.1% (w/v). The composition ratios of different hydrogels are shown in Table 1. The hydrogel was finally obtained by light (405 nm, 3 W/cm^2^) irradiation for 20 s.

**TABLE 1:** Composition ratios of different hydrogels

	GelMA (mg/ml)	OP3‐MA (mg/ml)	A‐CXCL9@PCEC (mg/ml)
5%GelMA	50	–	–
5%GelMA/OP3‐MA	50	0.6	–
10%GelMA/OP3‐MA	100	0.6	–
15%GelMA/OP3‐MA	150	0.6	–
10%GelMA/OP3‐MA/A‐CXCL9@PCEC	100	0.6	0.02

### 2.5 Characterization of the PCEC and A‐CXCL9@PCEC nanoparticles

The morphology of PCEC and A‐CXCL9@PCEC nanoparticles was observed by transmission electron microscope (TEM, Hitachi, H‐800). PCEC and A‐CXCL9@PCEC nanoparticles were first dispersed in deionized water by ultrasonic, and then added dropwise in copper mesh. The sample was then completely dried at room temperature and observed by TEM.

The hydrate diameter and particle size distribution of nanoparticles were observed by dynamic laser scatterometer (DLS, Malvern, Zeta Sizer Nano ZS).

### 2.6 Chemical structure characterization of GelMA and OP3‐MA

The chemical structure of GelMA and OP3‐MA was characterized by Fourier transform infrared spectrometer (FTIR, Inova‐500M, Varian, USA) and nuclear magnetic resonance spectrometer (NMR, VERTEX 70, Burke, Germany). For FTIR characterization, 5 mg of samples was first mixed with 30 mg of potassium bromide (KBr) and ground into powder, then the powder was pressed into a transparent flake and tested by FTIR. The scan range of the wavenumber was 4000–500 cm^−1^, and the resolution was 4 cm^−1^. As for NMR characterization, samples were dissolved in deuterated water (D_2_O), and the hydrogen spectrum was detected.

### 2.7 Characterization of Hydrogel

Rotational rheometer (Kinexus pro, Malvern, UK) was used to measure the rheological behavior of hydrogels. The 400 μl of hydrogel was used for each test. A flat rotor with a diameter of 25 mm was selected for the test. Time sweep sequence was performed at 25°C under fixed strain (1%). Frequency sweep was conducted in the range of 0.1–10 Hz under fixed strain (1%) at 25°C.

Morphology of the hydrogel was characterized by scanning electronic microscope (SEM, S‐3400, Hitachi, Japan). Hydrogels were first lyophilized under −80°C to remove water, and then fixed on copper sample stage using carbon conductive tape. The samples were spread with gold for 30 s before observation.

Compression test of hydrogel was tested by universal testing machine (ELF3200; Bose, USA). Hydrogels were pre‐prepared into a cylinder with a height of 6 mm and a diameter of 11 mm. Then samples were compressed using universal testing machine at a stable speed (0.05 mm/s). Compressed length and load were recorded by machine and the strain and stress were calculated by the following formula:

$$\text{Strain}\;\left(\%\right)=\frac{l_{0}-l_{1}}{l_{0}}\times 100\%$$

$$\text{Stress}=\frac{P}{A}\;\left(\mathrm{Pa}\right)$$

where l_0_ represents the initial height of sample, while l_1_ is the height of sample at different time point. P is the compression load, while A is the cross‐sectional area of hydrogel.

Equilibrium‐swelling ratio was measured by gravimetric method. Hydrogels were first lyophilized and weighted to obtain the initial weight (W_dry_). Immerse the dried hydrogel to PBS buffer (pH = 7.4) at 37°C, then take them out and weight at selected time point. The weight of hydrogel at different time point was recorded as W_swollen_. Equilibrium‐swelling ratio was calculated by the following formula:

$$\text{Equilibrium}-\text{swelling ratio}=\frac{W_{\text{swollen}}-W_{\mathrm{dry}}}{W_{\mathrm{dry}}\times 100\%}.$$

The degradation behavior with and without lysosome was also measured by gravimetric method. Hydrogels were first lyophilized and weighted to obtain the initial weight (W_0_). Then the hydrogels were immersed in PBS buffer (pH = 7.4) and 1000 U lysosome PBS solution separately. Finally, the hydrogel was taken out, lyophilized and weighted at selected time point. The weight of hydrogel at different time point was recorded as W_t_. Degradation ratio was calculated by the following formula:

$$\text{Degradation ratio}=\frac{W_{o}-W_{t}}{W_{0}\times 100\%}.$$

### 2.8 Cell compatibility of the hydrogel

#### 2.8.1 Cytotoxicity of hydrogel to rBMSCs

GelMA, OP3‐MA, and A‐CXCL9@PCEC solution were first filtered through 0.22 μm filter membrane to remove bacterial. Then 500 μl hydrogel was prepared in 12 well plates as described in Section 2.4. Rat bone marrow mesenchymal stem cells (rBMSCs) were cultured with DMEM containing 10% FBS and 1% penicillin–streptomycin solution. The rBMSCs in the logarithmic growth phase were trypsinized and resuspended, and 2 ml of cell suspension with a cell density of 2 × 10^4^ cells/ml was seeded on the surface of the hydrogel.

RBMSCs were co‐cultured with hydrogel for 1, 3, and 5 days in 5% CO_2_ incubator at 37°C. Cells at pre‐set time point were washed with PBS buffer for twice and incubated with 10% CCK‐8 solution for 1 h. The supernatant of cell‐hydrogel co‐culture system was collected, and the optical density (OD) value of supernatant at 450 nm was measured by microplate reader. Cell viability was calculated as following:

$$\text{Cell viability}=\frac{{\mathrm{OD}}_{\left(\text{materials}\right)}-{\mathrm{OD}}_{\left(\text{blank}\right)}}{{\mathrm{OD}}_{\left(\text{control}\right)}-{\mathrm{OD}}_{\left(\text{blank}\right)}}\times 100\%$$

Materials, blank, and control separately represent rBMSCs treated with different hydrogel, complete DMEM containing CCK‐8 without rBMSCs and rBMSCs treated with complete DMEM culture medium.

#### 2.8.2 Live/dead staining of rBMSCs co‐cultured with hydrogels

Calcein AM and propidium iodide (PI) was used for cell staining. Live cells can be stained with calcein AM to show green fluorescence, while dead cells stained with PI to show red fluorescence. RBMSCs were co‐cultured with hydrogel as described in Section 2.8.1 for 1, 3, and 5 days. Then the hydrogel was washed with PBS for three times and stained with calcein AM/PI following manufactures' direction. Images of live/dead staining were captured with inverted fluorescence microscope.

#### 2.8.3 Cytoskeleton staining of rBMSCs co‐cultured with hydrogels

The 500 μl of rBMSCs at cell density of 60,000/ml was seeded on GelMA‐based hydrogels and cultured at 37°C for 1, 3, and 7 days. Hydrogels were washed with PBS and fixed with 4% paraformaldehyde for 10–15 min at selected time. Then 0.5% TritonX‐100 was used to treated hydrogels for 5 min. After washed with PBS, TRITC Phalloidin working solution and DAPI were used to stain the cytoskeleton. Finally, the stained images were acquired by confocal laser microscopy.

### 2.9 Osteogenesis evaluation

After rBMSCs cultured with hydrogels leach liquor for 1, 3, and 5 days, the cells were stained with alkaline phosphatase (ALP) staining kit. After rBMSCs cultured with hydrogels leach liquor for 10 days, the cells were stained with alizarin red staining kit and mineralized nodules were captured by inverted microscope.

Immunofluorescence staining of osteogenic‐related proteins was also conducted. After co‐cultured with the hydrogel leach liquor, the rBMSCs cell slides were fixed with 4% paraformaldehyde, then the membrane was permeabilized with 0.3% Triton‐X, and then blocked with 5% BSA overnight. Then, the cells were incubated with primary antibodies against RUNX2, OCN, AKT and p‐AKT at room temperature for 1 h and then incubated with secondary antibody conjugated with fluorescent labels at room temperature for 1 h in dark.

### 2.10 In vivo femur defect rat model for osteogenic evaluation

#### 2.10.1 In vivo femur defect rat model

All animal experimental protocols have been reviewed and approved by the Animal Protection and Use Committee of The Eighth Affiliated Hospital of Sun Yat‐sen University (approval number: 2019d084). Thirty‐six female SD rats (200–220 g) were randomly divided into four groups (Blank, GelMA, GelMA/OP3‐MA, GelMA/OP3‐MA/A‐CXCL9@PCEC). Pentobarbital sodium (60 mg/kg) was used for intraperitoneal injection to anesthetize rats. Surgical instruments are preautoclaved. Hair on the right leg was shaved and exposed skin was disinfected by iodophor before surgical. Skin of the right femur was cut longitudinally by scalpel. Then muscle, ligament, and femur were separated by dental scraper to expose the distal side of the femur. Bone defect at the femur epiphysis with a diameter of 2.8 mm and a depth of 3 mm was subsequently created using an electric drill.^25^ The 20 μl of hydrogel presolution was then added on the bone defect and crosslinked by blue light for 20 s. Control group was treated by 20 μl of PBS. Muscle and skin were suture the in turn using 0‐3 absorbable sutures, and surgical site was finally disinfected with iodophor. Penicillin was injected intramuscularly within 3 days after surgery to avoid infection.

Femur was taken out after treating for 7, 14, and 28 days and fixed on 4% paraformaldehyde for 24 h. The femur was decalcified with EDTA decalcification solution for 1 month before paraffin embedding. Injured femur was cut into 5 μm thick slices, and then pathologically stained.

#### 2.10.2 Micro‐CT scanning

Femur without soft tissue was fixed on 4% paraformaldehyde and scanned with micro‐CT (MCT‐Sharp, Zhongke Kaisheng, China; scanning voltage: 70 kV; Voxel size: 20 μm). 3D reconstruction and quantitative analysis were conducted using the sagittal image of the distal femur. The region of interest (ROI) was defined as 900 μm (45 consecutive images) of the proximal end of the epiphysis of the distal femur.

#### 2.10.3 Pathological staining and immunofluorescence staining

Femurs were decalcified, embedded in paraffin, and cut into 4 μm slices. H&E, Masson and TRAP staining were performed according to standard protocols.^26^ Angiogenesis effect was identified by immunofluorescence‐labeled α‐SMA. OCN, OPN, Col‐I, and TGF‐β1 were conducted through immunohistochemical labeling.

### 2.11 Statistical analysis

Each group of samples in the experiment contains at least three parallel samples, and the results were shown as average and standard deviation. The significance was analyzed using Graphpad Prism 7.0. One‐way analysis of variance (ANOVA) was performed to evaluate the significance of the experimental data. The statistical significance was *p < 0.05, **p < 0.01, and ***p < 0.001.

## 3 RESULTS

### 3.1 Physicochemical structure characterization of hydrogel and nanomaterials

**SCHEME 1:**
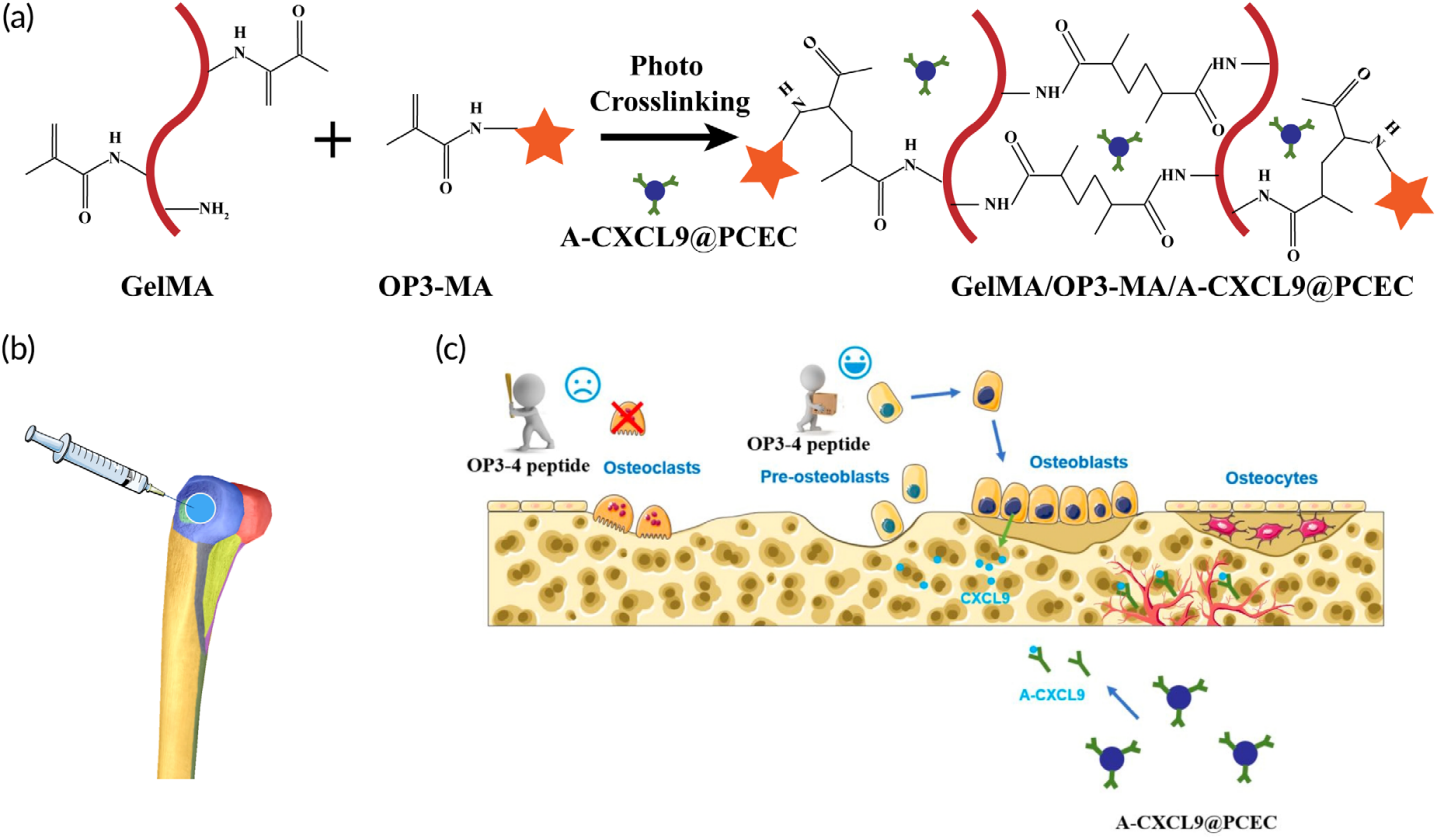
Schematic diagram of the preparation and action process of the hydrogel. (a) Schematic diagram of the gelation process of hydrogels; (b) hydrogel injection into femoral injury cavity; (c) schematic representation of the role of OP3‐4 and A‐PCEC: The OP3‐4 peptide released from hydrogel prevents osteoclasts formation and promotes pre‐osteoblasts to become osteoblasts; A‐CXCL9 released in the same time to neutralize the excess CXCL9 secreted by osteoblast to promote angiogenesis.

The hydrogel was obtained by photo‐initiated free‐radical polymerization between GelMA and OP3‐MA, which can sustain release of OP3‐4 cyclic peptide^9^ to block the activation of NF‐κB signaling pathway thus inhibit osteoclast formation and bone resorption.^27^ Amphiphilic block copolymers PCEC can spontaneously assemble into nano micelle and carry the anti‐CXCL9 antibody (A‐CXCL9)^21^, ^22^ through electrostatic adsorption, and let anti‐CXCL9 antibody sustain release to the surrounding environment to promote the angiogenesis effect and bone formation (Scheme 1).

**FIGURE 1:**
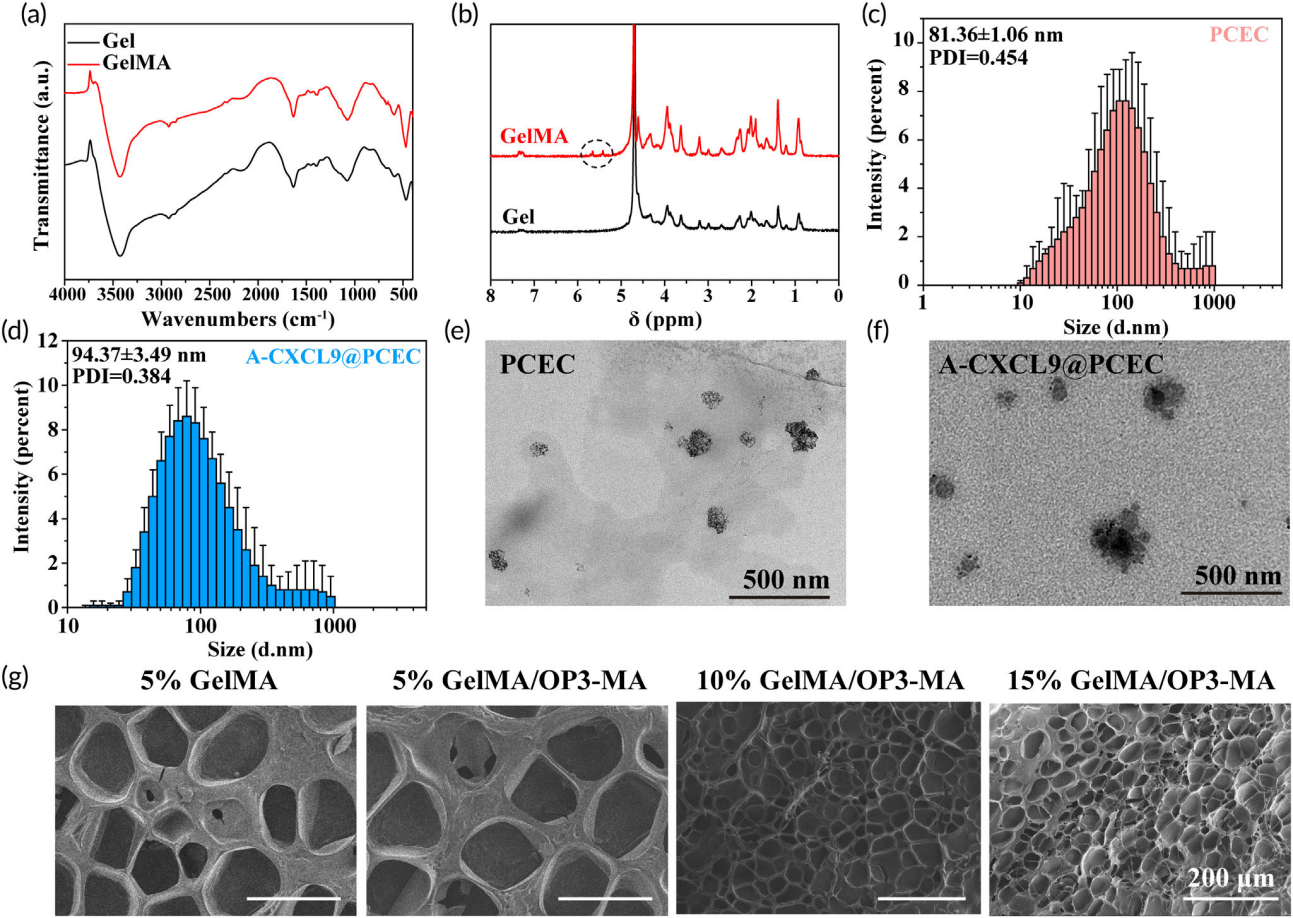
Physicochemical structure characterization of hydrogel and nanomaterials. (a) Fourier transform infrared (FTIR) and (b) ^1^H NMR spectra of Gelatin (Gel) and GelMA; size distribution and PDI of (c) PCEC and (d) A‐CXCL9@PCEC nanoparticles; transmission electron microscopeTEM image of (e) PCEC and (f) A‐CXCL9@PCEC nanoparticles, scale bar = 500 nm; (g) Morphology of lyophilized hydrogel with different composition, scale bar = 200 μm

Figure 1 displays the typical physicochemical and microstructure characterization of hydrogels and nano micelles. Chemical structure of GelMA was analyzed by FTIR and ^1^H NMR. The difference in the FTIR spectra of GelMA compared to Gelatin (Gel) was not obvious because both containing a large number of amide bonds. The absorption peak of GelMA at 592 cm^−1^ was stronger than that of Gel, which may be due to more amide bonds in GelMA, and the absorption peak of the amide VI band appears enhanced (Figure 1a). The two newly appeared peaks at δ 5.66 and δ 5.43 ppm on GelMA were linked to acrylic protons,^11^ indicated successful synthesis of GelMA (Figure 1b).

The size distribution of PCEC and A‐CXCL9@PCEC nanoparticles was 81.36 ± 1.06 nm and 94.37 ± 3.49 nm, respectively (Figure 1c,d). PCEC had negative zeta potential of −32.70 ± 0.61 mV, after carrying the positive A‐CXCL9, the zeta potential of A‐CXCL9@PCEC nano micelles increased to −7.02 ± 0.06 mV. Both PCEC and A‐CXCL9@PCEC had a concentrated particle‐size distribution, and the diameter of PCEC slightly increased after loading A‐CXCL9. Figure 1e,f shows the typical spherical morphology of PCEC and A‐CXCL9@PCEC. The morphology of the hydrogel observed by SEM showed that as the concentration of GelMA increased, the degree of crosslinking of the hydrogel also increased and the pore size decreased (Figure 1g). The average pore size of different hydrogels was shown in Figure S1. It could be noticed that though OP3‐4 was previously modified with methacrylic bond (OP3‐MA) and crosslinked with GelMA during the hydrogel formation process, the pore size of 5% GelMA and 5% GelMA/OP3‐MA did not show obvious difference (about 160 μm), which may owe to the less mass fraction of OP3‐MA (only 0.06%).

### 3.2 Mechanical and degradation behavior of hydrogel

**FIGURE 2:**
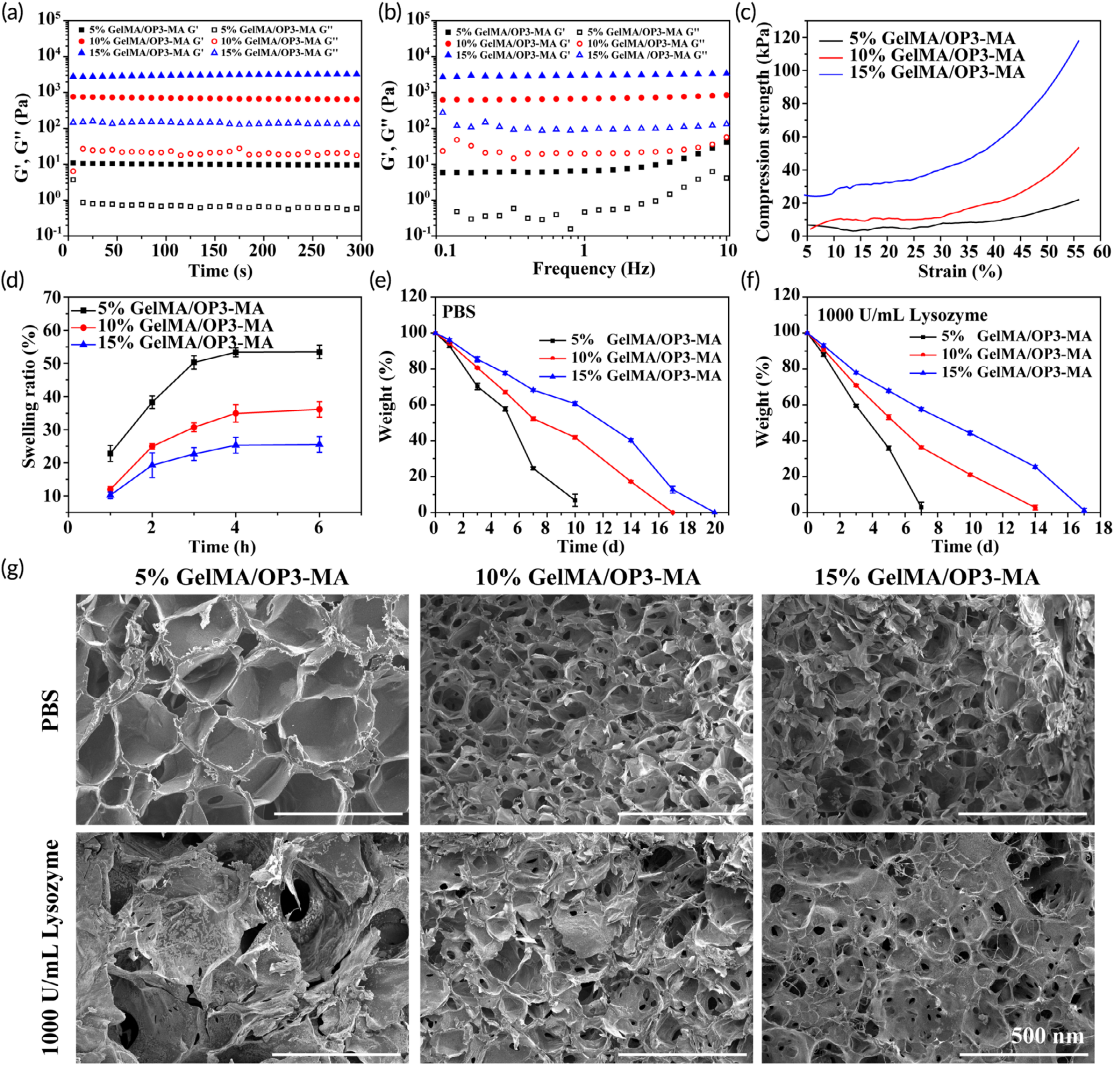
Rheological, compression and degradation behavior of GelMA/OP3‐MA hydrogel. (a) Time‐sweep and (b) frequency‐sweep sequence of GelMA/OP3‐MA hydrogel; (c) Stress–strain curve of GelMA/OP3‐MA hydrogel; (d) in vitro swelling behavior of GelMA/OP3‐MA hydrogel; in vitro degradation behavior of GelMA/OP3‐MA in (e) PBS and (f) 1000 U/ml lysozyme PBS solution; (g) morphology of degraded GelMA/OP3‐MA hydrogel after being degraded in PBS and 1000 U/ml lysozyme PBS solution for 5 days

Figure 2 shows the rheological, compression, and degradation behavior of GelMA/OP3‐MA hydrogel. The solution of GelMA/OP3‐MA containing LAP was initiated with blue light (405 nm) and formed hydrogel within 20 s. GelMA/OP3‐MA hydrogel with different GelMA mass fraction all showed steady storage modulus (*G*') and loss modulus (*G*") in time sweep sequence, and *G*' > *G*", which means the hydrogel had totally formed (Figure 1a). As the mass fraction of GelMA increased, the linear viscoelastic zone of the GelMA/OP3‐MA hydrogel gradually increased, and the 15% GelMA/OP3‐MA hydrogel exhibited linear viscoelasticity between 0.1 and 10 Hz (Figure 2b). Stress–Strain curve is shown in Figure 2c. Similar to the trend of *G*', the compression strength of GelMA/OP3‐MA hydrogel also showed a positive correlation with the GelMA mass fraction. Compressive strength of GelMA/OP3‐MA hydrogel with increased GelMA mass fraction was 21.95, 51.31, and 117.74 kPa, respectively.

In vitro swelling and degradation behavior showed that the 15% GelMA/OP3‐MA hydrogel had the smallest swelling ratio 25.54% ± 2.38% and longest degradation time both in PBS and lysozyme PBS solution (Figure 2d–f). Owing to the highest crosslinking density of 15% GelMA/OP3‐MA hydrogel, the swelling ratio of 15% GelMA/OP3‐MA hydrogel was nearly half of 10% GelMA/OP3‐MA hydrogel (36.12% ± 2.36%) and 5% GelMA/OP3‐MA hydrogel (53.45% ± 2.02%); 15% GelMA/OP3‐MA hydrogel also behaved slowest degradation time in the in vitro degradation test with or without lysozyme; 15% GelMA/OP3‐MA hydrogel completely degraded on 20 days in PBS and 17 days in PBS containing 1000 U/ml lysozyme. A faster degradation rate showed in the 5% GelMA/OP3‐MA hydrogel and 10% GelMA/OP3‐MA hydrogel, which had relatively lower crosslinking density. While 5% GelMA/OP3‐MA hydrogel was completely degraded in 7 days. SEM images of the degraded hydrogel on Day 5 had also been shown in Figure 2g. The surface morphology of the hydrogel degraded in lysozyme solution shows a collapsed pore structure compared to the hydrogel degraded in PBS. This phenomenon is most obvious in 5% hydrogels, indicating that the enzyme environment can be significantly accelerated to destroy the cross‐linked structure of the hydrogel, thereby accelerating the degradation of the hydrogel.^28^

The above results can indicate that the mechanical properties and degradation time of the hydrogels we prepared are adjustable. The migration of mesenchymal stem cells (MSCs) is a key factor in tissue regeneration, and the crosslinking density of the hydrogel has a great influence on the migration of MSCs. Although the migration of cells on medium‐modulus hydrogels (100–1000 Pa) is not as good as that on low‐modulus hydrogels (≈100 Pa),^29^ in order to avoid the hydrogels degrading too quickly in vivo and unable to exert long‐term effects, we finally chose 10% GelMA/OP3‐MA hydrogel for subsequent applications.

### 3.3 Cell compatibility of hydrogel

**FIGURE 3:**
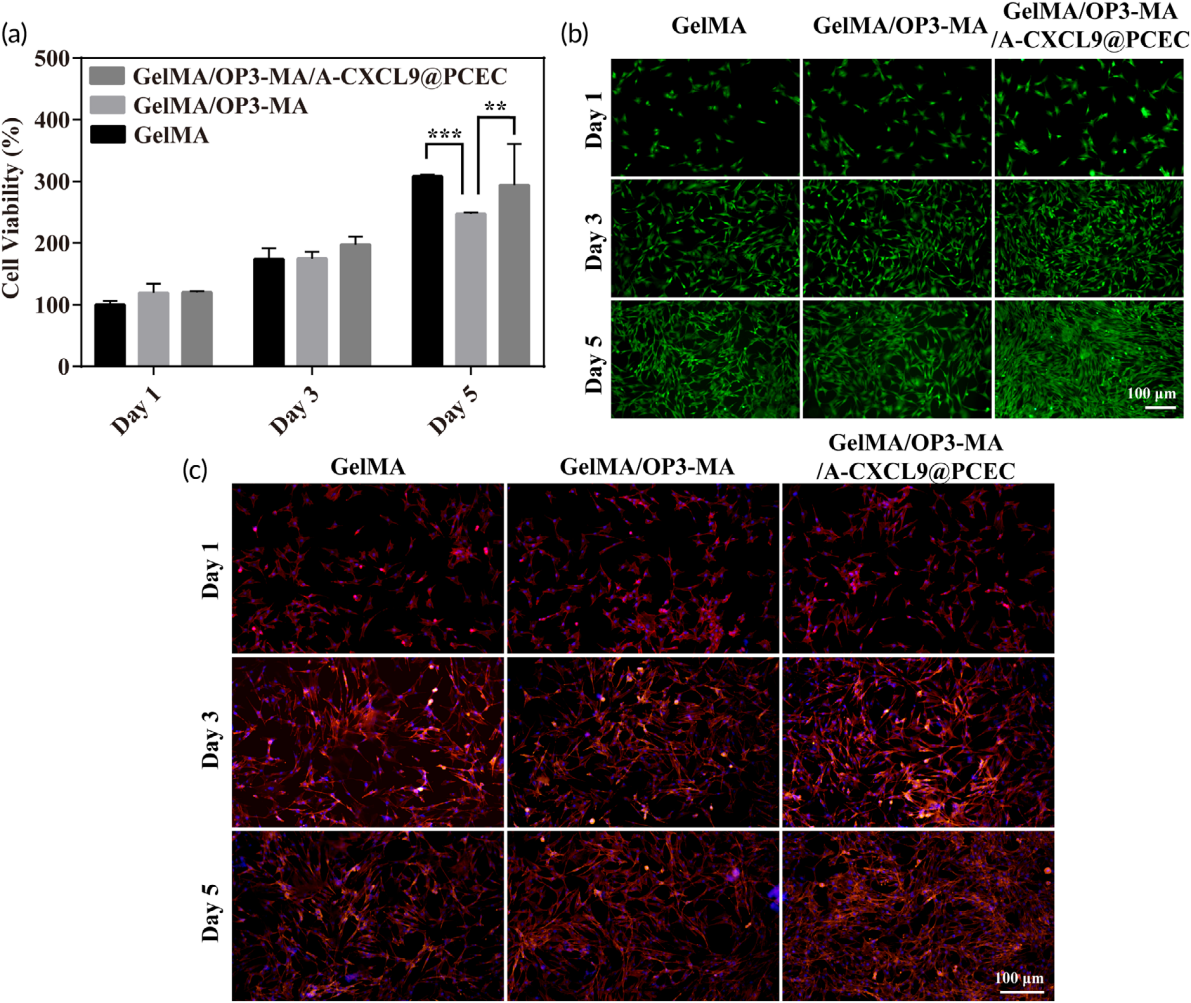
Cell compatibility of hydrogels. (a) Viability of rat bone marrow mesenchymal stem cells (rBMSCs) co‐cultured with hydrogels detected by CCK‐8 assay; (b) Live/dead staining of rBMSCs co‐cultured with hydrogels (red: dead cells; green: live cells.); (c) Cytoskeleton of 3D cultured rBMSCs on hydrogels stained with phalloidin

The rBMSCs was co‐cultured with hydrogels to evaluate the cell compatibility. As shown in Figure 3a, the cell viability increased with the extension of the culture time, showing good cell proliferation. The control group GelMA showed the best effect of promoting cell proliferation, and the cell viability on Days 1, 3, and 5 was 100 %± 6.46%, 174.16% ± 17.61%, and 308.08% ± 2.97%, respectively. This is because gelatin itself contains a large number of short RGD peptides, which can obviously promote cell adhesion and proliferation.^19^ Therefore, GelMA has been used in the construction of tissue engineering materials for a long time with excellent biocompatibility.^19^, ^20^ When OP3‐MA cyclic peptide was co‐crosslinked with GelMA, during the first and third days of co‐culture, the cell survival rate did not show a significant difference compared with the other two groups. However, after 5 days of culture, the cell proliferation rate of GelMA/OP3‐MA hydrogel slowed down, which was significantly lower than the other two groups, and the cell survival rate was 247.42% ± 2.30%. After adding A‐CXCL9@PCEC, the rBMSCs showed a higher proliferation effect than GelMA/OP3‐MA hydrogel, and the cell survival rate on Day 5 was 294.08% ± 66.94%, which was not significantly different from GelMA hydrogel. Figure 3b shows the live/dead staining of rBMSCs. Although the rBMSCs showed a low proliferation rate in the GelMA/OP3‐MA hydrogel, there were no obvious dead cells during the whole culturing period, indicating that the hydrogel was not cytotoxic, but the addition of OP3‐MA and A‐CXCL9@PCEC would affect the proliferation of cells.^30^

Phalloidin stains the actin in the microfilaments in the cytoskeleton and shows red fluorescence.^31^ From the staining of the cytoskeleton in Figure 3c, it could be seen that the rBMSCs cultured on GelMA/OP3‐MA hydrogel and GelMA/OP3‐MA/A‐CXCL9@PCEC hydrogel presented a fully unfolded fusiform structure faster after the first day of culture and showed longer microfilaments, indicating that the rBMSCs could adhere well to the GelMA hydrogels scaffold. This result was consistent with the biological effect of GelMA.^20^ When the culture time increased to 5 days, all rBMSCs grown on the hydrogels show a spreading cytoskeleton, and the microfilament junctions between cells were denser, which indicated that the rBMSCs on the hydrogel can form a tightly connected structure. This will help maintain tissue integrity and strengthen communication between cells.

### 3.4 In vitro A‐CXCL9 release and promoting osteogenesis

A‐CXCL9 only binds to PCEC through weak electrostatic interaction and is encapsulated in the hydrogel. With the degradation and diffusion of the hydrogel, A‐CXCL9 can be gradually released into the surrounding environment to promote bone formation. Figure S2 showed the standard curve of A‐CXCL9 measured by ELISA. Totally 2 μg of A‐CXCL‐9 was carried in 1 ml of hydrogel. By testing the A‐CXCL9 released from the hydrogel, we found that the release of A‐CXCL9 reached equilibrium after the 7th day, and the final release amount could reach to 58.78% (Figure S3).

**FIGURE 4:**
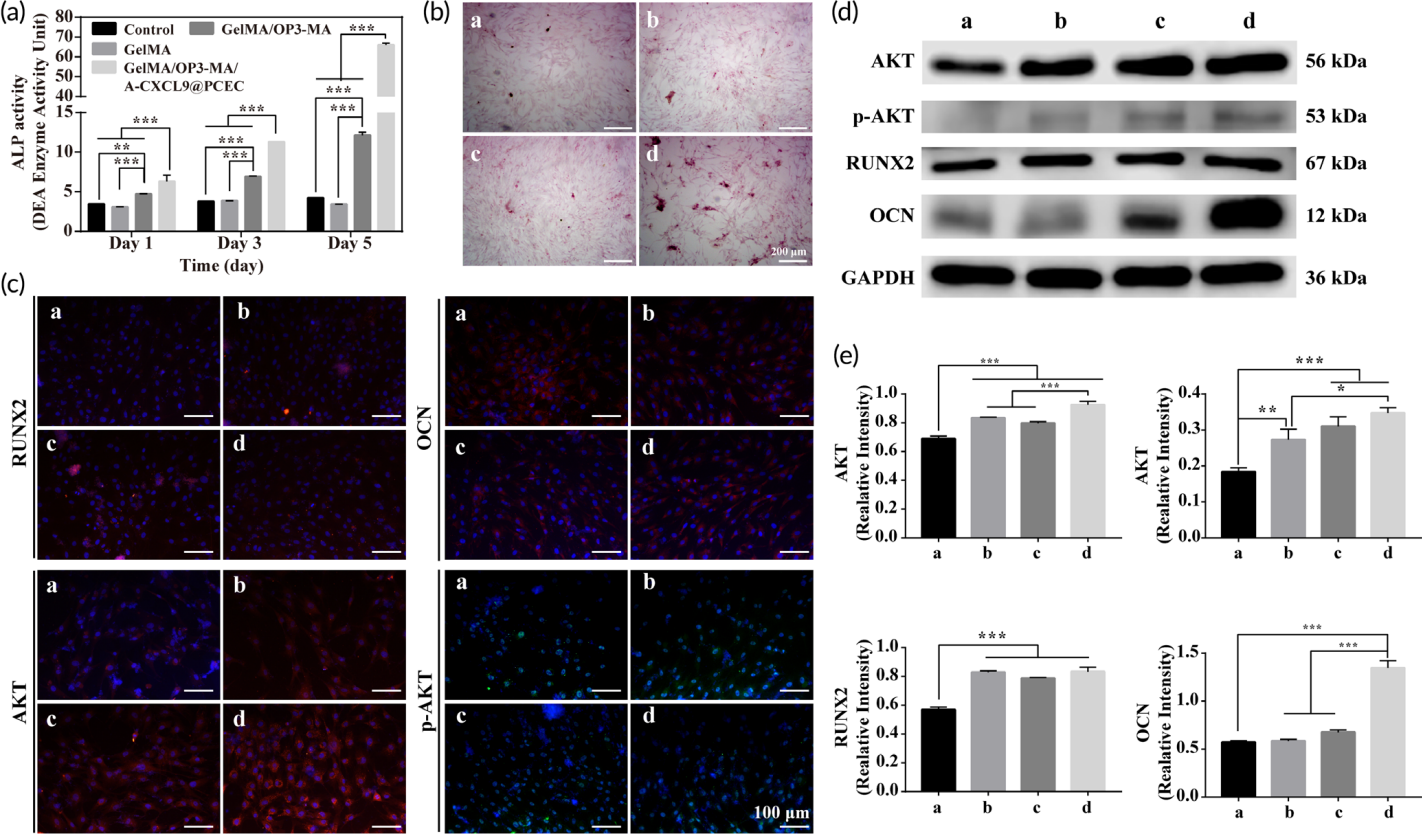
In vitro bone formation ability and signaling pathway. (a) ALP activity of rBMSCs co‐cultured with hydrogels for 1, 3, and 5 days; (b) Alizarin Red S of rBMSCs cultured with hydrogels leach liquor for 10 days for detection of in vitro Ca^2+^ deposition; scale bar = 200 μm; (c) Immunofluorescence staining of osteogenic markers OCN, RUNX2 AKT and p‐AKT; (d) Western blot and (e) quantitatively analysis of OCN, RUNX2 AKT and p‐AKT. (a: Control; b: GelMA; c: GelMA/OP3‐MA; d: GelMA/OP3‐MA/A‐CXCL9@PCEC); scale bar = 100 μm

The in vitro promoting osteogenic differentiation ability of hydrogel was detected by co‐cultured with rBMSCs. ALP activity was used to indirectly quantify the ability of rBMSCs to differentiate into osteoblasts.^32^ Figure 4a shows that ALP activity all increased for 5 days of co‐culturing, but only in GelMA/OP3‐MA and GelMA/OP3‐MA/A‐CXCL9@PCEC hydrogels there were obviously changes. In the 1, 3 and 5 days of co‐culture, the ALP activity of GelMA/OP3‐MA and GelMA/OP3‐MA/A‐CXCL9@PCEC hydrogels was significantly higher than control and GelMA group, and the GelMA/OP3‐MA/A‐CXCL9@PCEC hydrogel has the highest ALP activity. Alizarin red staining reflects the deposition of calcium ions in cells, which is an important characterization for the study of bone mineralization.^33^ By staining the rBMSCs cultured on the hydrogel with Alizarin Red, we found that the rBMSCs in groups GelMA/OP3‐MA and GelMA/OP3‐MA/A‐CXCL9@PCEC showed more pink calcium salt deposition, indicating that these two hydrogels can better promote bone mineralization and complete the last step of bone formation (Figure 4b).

In order to clarify the relevant mechanism of the above results, we performed immunofluorescence staining on the expression of bone formation‐related genes in the rBMSCs, and the results were shown in Figure 4c. ALP and osteocalcin (OCN) are two classic markers of osteogenic differentiation. The expression of OCN and ALP has been confirmed to be the strongest in GelMA/OP3‐MA/A‐CXCL9@PCEC hydrogel. RNUX2 is the upstream target of ALP and OCN.^34^ We found that the expression of RUNX2 was stronger in GelMA/OP3‐MA hydrogel. Moreover, the high expression of AKT and p‐AKT also appeared in GelMA/OP3‐MA and GelMA/OP3‐MA/A‐CXCL9@PCEC hydrogels (Figure 4c).

Quantitative statistics and analysis on the expression of AKT, p‐AKT, RUNX2, and OCN were then performed (Figure 4d,e). We found that hydrogels containing OP3‐4 polypeptides could significantly increase the expression of AKT, P‐AKT and OCN, while the hydrogel‐treated group that simultaneously released A‐CXCL9 and OP3‐4 had the highest expression of OCN, indicating that A‐CXCL9 can synergy working with OP3‐4 to achieve higher expression of osteogenesis‐related proteins. From the results of immunofluorescence and western blot, we speculate that OP3‐MA and OP3‐MA/A‐CXCL9 may be through activating the AKT‐RUNX2‐ALP pathway and ultimately promote osteogenic differentiation.

### 3.5 The GelMA/OP3‐MA/A‐CXCL9@PCEC hydrogel promotes bone regeneration in vivo

**FIGURE 5:**
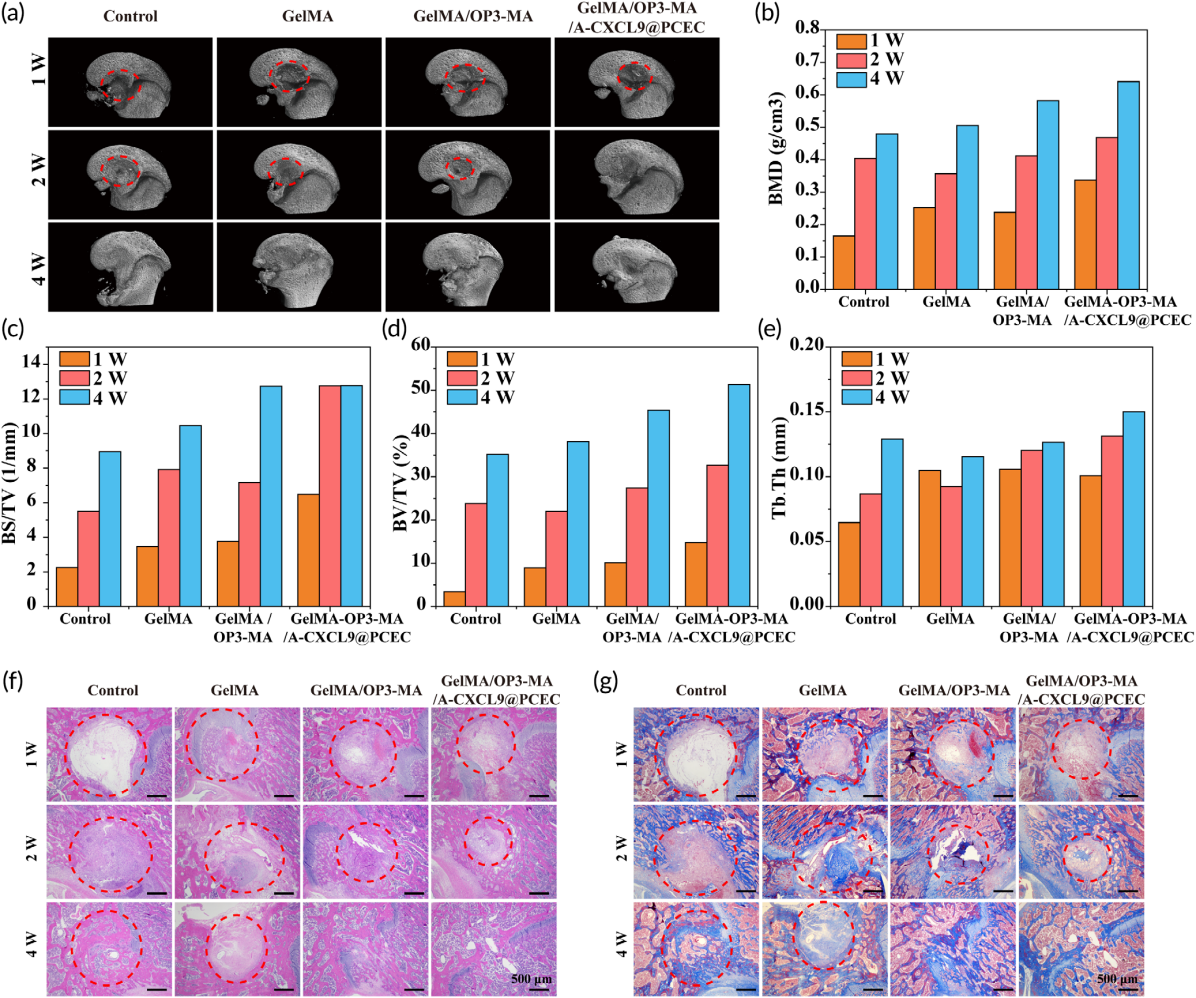
The GelMA/OP3‐MA/A‐CXCL9@PCEC hydrogel promotes femur regeneration in vivo. (a) Micro‐CT images of femur defect treated with different hydrogels obtained 1, 2, and 4 weeks after implantation; (b) bone mineral density (BMD), (c) bone surface/bone volume (BS/TV), (d) bone volume fraction (BV/TV) and (e) trabecular thickness (Tb/Th) in different groups; (f) H&E and (g) Masson staining of the femur defect site; scale bar = 500 μm. The red circle represents the damage area.

After the GelMA/OP3‐MA/A‐CXCL9@PCEC, hydrogel was prepared and the promoting osteogenic differentiation ability had been demonstrated, we conducted round femur defect rat model to evaluate the bone regeneration in vivo. Micro‐CT data presented in Figure 5a showed that more bone formed in GelMA/OP3‐MA/A‐CXCL9@PCEC hydrogel treated group. After 1 week of treatment, the 3D reconstruction CT images can clearly observe the annular defect in the middle of the femur. The CT images after treating 2 weeks showed that there was new bone formation in the defect parts of the GelMA/OP3‐MA hydrogel and GelMA/OP3‐MA/A‐CXCL9@PCEC hydrogel‐treated groups and basically completely repaired after 4 weeks. GelMA/OP3‐MA/A‐CXCL9@PCEC hydrogel‐treated group showed highest bone mineral density (BMD), bone surface/tissue volume (BS/TV). BV/TV, and trabecular thickness (Tb.Th), which represents the fastest bone regeneration and the best quality (Figure 5b–e).

The results of H&E and Masson staining further showed the healing effect of the bone tissue at the injury site. After 1 week of treatment, the control group was able to observe obvious tissue defects without any tissue growth. In the hydrogel‐treated group, the tissues began to grow inward gradually from around, and a small amount of collagen deposition appeared. After the second week of treatment, the cavity was filled with new tissue, and GelMA/OP3‐MA/A‐CXCL9@PCEC hydrogel‐treated group showed denser collagen deposition. On the 4th week, GelMA/OP3‐MA/A‐CXCL9@PCEC hydrogel‐treated group was almost completely repaired, the collagen structure was denser and closer to normal tissue (Figure 5c,d). The histological staining was consistent with the CT images, indicating that the GelMA/OP3‐MA/A‐CXCL9@PCEC hydrogel‐treated group can promote bone tissue regeneration faster, and the bone tissue structure was denser, and close to normal bone tissue.

### 3.6 The GelMA/OP3‐MA/A‐CXCL9@PCEC hydrogel inhibits osteoclast differentiation and promotes vascularization

**FIGURE 6:**
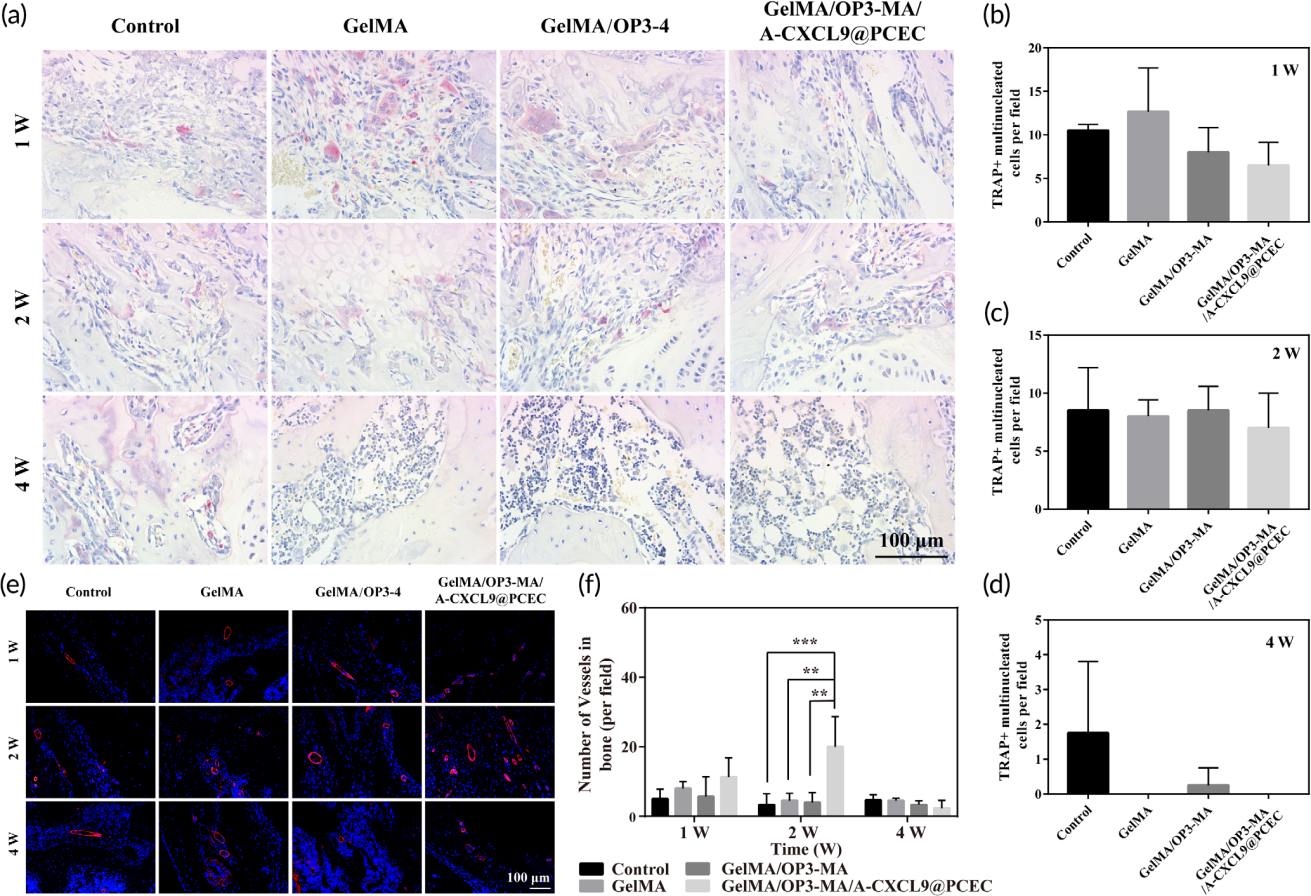
The assessment of inhibition of osteoclast activation and promotion of vascularization. (a) TRAP staining of bone tissue at different time points; Quantitively analysis of osteoclasts at (b) 1, (c) 2, and (d) 4 weeks; (e) Immunofluorescence staining image of α‐SMA in defect femur; (f) quantitatively analysis of blood vessels from immunofluorescence staining of α‐SMA; scale bar = 100 μm

OP3‐4 cyclic peptide can bind to nuclear factor κB receptor activator ligand (RANKL) to inhibit osteoclast activation and promote osteogenic differentiation. We performed TRAP staining on the femur to specifically characterize the differentiation of osteoclasts (Figure 6a). During the first week of treatment, obvious osteoclast formation appeared around the injured area in the control group and GelMA‐treated group, indicating that the injury led to increased differentiation of surrounding osteoclasts and slowed the rate of healing (Figure 6b). In contrast, the number of osteoclasts in the OP3‐4 cyclic peptide released hydrogel (GelMA/OP3‐MA and GelMA/OP3‐MA/A‐CXCL9@PCEC hydrogels) after 1 week of treatment was lower than that of the control and the GelMA group, indicating that the OP3‐4 peptide could inhibit the osteoclasts (Figure 6b). Two weeks after operation, the number of osteoclasts in the control group and GelMA group was less than that in the first week (Figure 6c). However, osteoclasts were still observed in the control group 4 weeks after operation, and there were almost no osteoclasts in the GelMA/OP3‐MA/A‐CXCL9@PCEC group 4 weeks after operation (Figure 6d). This difference indicates that OP3‐4 peptide can inhibit the formation of osteoclasts for a long time, and when used in combination with A‐CXCL9, it can better promote osteogenesis.

For the formation of bone tissue, in addition to inhibiting the formation of osteoclasts, another important stage is the vascularization of bone tissue.^35^ CXCL9 can be secreted by osteoblasts, interact with vascular endothelial growth factor (VEGF), and prevent VEGF from binding endothelial cells to form blood vessels.^16^ Therefore, excessive CXCL9 secreted by osteoblasts can inhibit the formation of blood vessels and slow down the healing process of bone tissue. We used α‐SMA fluorescent labeling of tissue neovascularization at different stages of bone regeneration, and the results were shown in Figure 6b. One week after operation, although neovascularization was observed in each group, there was no significant difference. It is worth noting that in the second week, the GelMA/OP3‐MA/A‐CXCL9@PCEC treatment group had obvious blood vessel formation, which was almost 4‐fold higher than the other groups (Figure 6c). Four weeks after operation, the number of new blood vessels decreased, owing to the healing tissue and decreased neovascularization rate.

### 3.7 The GelMA/OP3‐MA/A‐CXCL9@PCEC hydrogel promotes the expression of osteogenic proteins and the deposition of type I collagen

**FIGURE 7:**
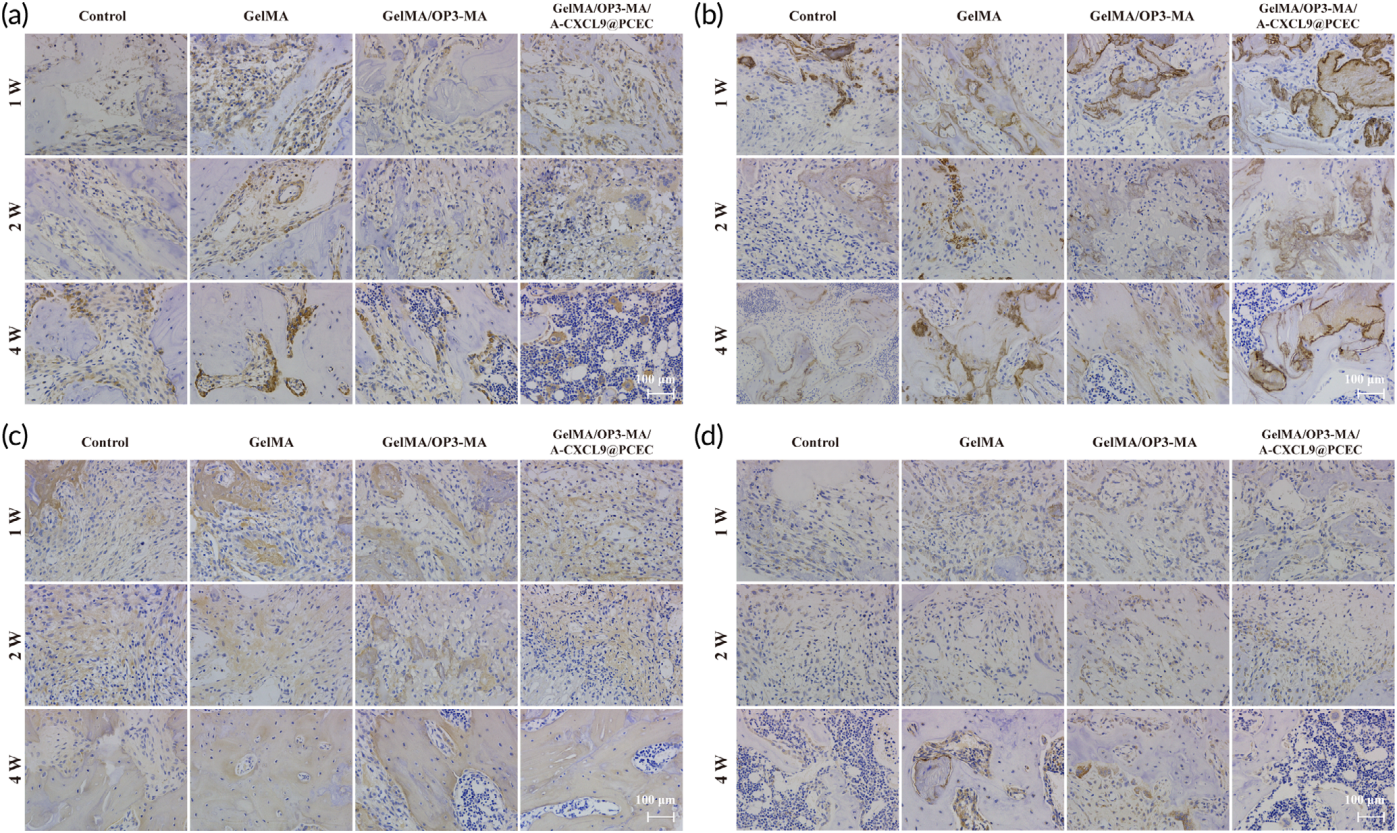
Immunohistochemical staining of osteogenesis‐related proteins and cytokines. Immunohistochemical staining of (a) OCN; (b) OPN; (c) Col‐I and (d) TGF‐β1; Scale bar = 100 μm

Immunohistochemical staining of bone tissue at different times after surgery was performed to evaluate the expression of osteogenesis‐related proteins. The expression of osteogenesis‐related proteins OCN and osteopontin (OPN) in the regenerated cancellous bone increased with the prolongation of treatment time. And the GelMA/OP3‐MA/A‐CXCL9@PCEC hydrogel treatment group had the highest expression (Figure 7a,b). Col I is the major structural protein of the extracellular matrix of bone and a representative marker of osteogenic differentiation. One to two weeks after operation, the collagen showed a random interweaving arrangement, which was a typical woven bone structure. Collagen fibers aligned in parallel 4 weeks after operation, forming a lamellar bone structure. The GelMA/OP3‐MA hydrogel and GelMA/OP3‐MA/A‐CXCL9@PCEC hydrogel treatment groups had faster Col I deposition at the first 2 weeks of treatment and more collagen fibers in the lamellar bone after 4 weeks (Figure 7c).

High expression of transforming growth factor β (TGF‐β1) was also observed in osteoblasts in the GelMA/OP3‐MA hydrogels treated group. TGF‐β1 was closely related to osteogenic differentiation, and up‐regulation of TGF‐β1 expression can significantly promote osteogenic differentiation (Figure 7d). The above results indicated that OP3‐4 polypeptide played a significant role in promoting osteogenic differentiation in the process of bone formation. At the same time, under the action of angiogenesis‐promoting factor A‐CXCL9, the expressions of osteogenesis‐related proteins OCN, OPN, and Col I were further increased.

## 4 DISCUSSION

The repair of damaged bone tissue involves two important issues: the balance of osteoblasts and osteoclasts^4^ and the process of vascularization.^36^ However, previous studies have found that CXCL9 secreted by osteoblasts binds endogenous VEGF and prevents VEGF from binding to endothelial cells, thereby interfering with the process of bone vascularization.^16^ Therefore, the coordination of the three factors of osteoblasts, osteoclasts, and angiogenesis has become a novel strategy to promote bone regeneration. The OP3‐4 polypeptide, as a RANKL‐binding peptide,^37^, ^38^ can inhibit the activation of osteoclasts^12^ while promoting the differentiation of osteoblasts,^15^ reconciling the first two therapeutic factors. To reconcile the third factor, the process of vascularization, we designed PCEC nanoparticles to simultaneously release anti‐CXCL9 antibody (A‐CXCL9) to neutralize excess CXCL9 secreted by osteoblasts.^16^, ^17^ Under the constraints of the free diffusion of GelMA hydrogel and the electrostatic interaction of PCEC, the sustained release of A‐CXCL9 was close to 60% after 8 days, and the release profile basically reached equilibrium. When the hydrogel was gradually degraded, the remaining A‐CXCL9 will be gradually released into the surrounding environment, continuously improving the inhibition of vascularization.^16^, ^17^

The entire coordination process was achieved by GelMA‐based hydrogels, which have good biocompatibility and degradability and are widely used in the repair of hard tissues such as bone and cartilage.^9^ Through in vitro toxicity assessment, we confirmed that the GelMA‐based hydrogel has no cytotoxicity and can well support the growth of rBMSCs. Moreover, the in vitro degradation results show that GelMA hydrogel can be completely degraded by hydrolysis and enzymatic hydrolysis and will not remain as foreign matter in the body for a long time.

In vitro regulation experiments of GelMA/OP3‐MA and GelMA/OP3‐MA/A‐CXCL9@PCEC hydrogels on rBMSCs showed higher ALP expression, significant inhibition of osteoclast differentiation and higher RUNX2 and AKT expression level, showed that the hydrogel may be through activating the AKT‐RUNX2‐ALP pathway, and ultimately promote osteogenic differentiation. A femur defect model was further performed to validate the therapeutic effect of GelMA/OP3‐MA/A‐CXCL9@PCEC hydrogel. We found that through this coordinated osteoblast, osteoclast and angiogenesis pathway, bone regeneration process can be accelerated. The expression of osteogenesis‐related proteins Col I, OCN, and OPN was significantly increased, and highest number of blood vessels can be observed after 2 weeks of GelMA/OP3‐MA/A‐CXCL9@PCEC hydrogel treatment. This indicates that the degree of vascularization in the first 2 weeks of treatment is likely to determine the speed and quality of bone healing in the later period. Because a high degree of vascularization is more conducive to the transfer of nutrients and can promote the growth of bone tissue inside the injury.^4^, ^5^, ^35^

## 5 CONCLUSION

In this study, we established a novel bone regeneration pathway that simultaneously coordinates osteoblast, osteoclast, and vascularization processes. Co‐crosslinking of OP3‐4 polypeptides by GelMA hydrogel promotes osteogenic differentiation and inhibits osteoclast activation. At the same time, PCEC nanoparticles were loaded with A‐CXCL9 to neutralize endogenous CXCL9 and promote vascularization. The results of promoting osteogenic differentiation in vitro and in vivo showed that the GelMA/OP3‐MA/A‐CXCL9@PCEC hydrogel that simultaneously modulated these three behaviors had the best osteogenic differentiation results, and the vascularization level was the highest at the second week. The GelMA/OP3‐MA/A‐CXCL9@PCEC hydrogel had fastest bone repair rate, higher collagen deposition amount and expression of osteogenesis‐related proteins OCN and OPN. This novel scaffold promotes bone regeneration by simultaneously inhibiting osteoclast activation and increasing vascularization, and our study provides a strategy for the bone defect.

## AUTHOR CONTRIBUTIONS

**Peng Luo:** Investigation (lead); methodology (lead); writing – original draft (equal). **Jiarui Fang:** Conceptualization (equal); data curation (equal); formal analysis (equal); writing – original draft (equal). **Dazhi Yang:** Conceptualization (lead); project administration (equal); resources (equal); supervision (equal); validation (equal); visualization (equal). **Lan Yu:** Data curation (equal); formal analysis (equal); methodology (equal). **Houqing Chen:** Conceptualization (equal); formal analysis (equal); investigation (supporting); methodology (supporting); software (equal). **Changging Jiang:** Funding acquisition (equal); project administration (equal); supervision (equal); writing – review and editing (equal). **Shuo Tang:** Project administration (equal); resources (equal); supervision (equal); validation (equal); writing – review and editing (equal). **Tao Zhu:** Project administration (equal); resources (equal); supervision (equal). **Rui Guo:** Project administration (equal); resources (equal); supervision (equal).

## CONFLICT OF INTERESTS

The authors declare no competing financial interest.

### PEER REVIEW

The peer review history for this article is available at https://publons.com/publon/10.1002/btm2.10414.

## Supporting information


**Figure S1** Average pore size of hydrogel.
**Figure S2.** Standard curve of A‐CXCL9.
**Figure S3.** In vitro release behavior of A‐CXCL9.

## Data Availability

The data that support the findings of this study are available from the corresponding author upon reasonable request.
