# Supplementary material for: OP3‐4 peptide sustained‐release hydrogel inhibits osteoclast formation and promotes vascularization to promote bone regeneration in a rat femoral defect model
Source: Bioeng Transl Med. 2022 Oct 11;8(2):e10414. doi: 10.1002/btm2.10414 (PMC10013759; doi:10.1002/btm2.10414)
Supplement: Supplementary file 1 — Figure S1 Average pore size of hydrogel. Figure S2. Standard curve of A‐CXCL9. Figure S3. In vitro release behavior of A‐CXCL9. [file BTM2-8-e10414-s001.docx]

**OP3-4 Peptide Sustained-release Hydrogel** **Inhibits Osteoclast Formation and Promotes Vascularization to Promote Bone Regeneration in a Rat Femoral Defect Model**

Peng Luo^1,#^, Jiarui Fang^1,#^, Dazhi Yang^2,#^, Lan Yu^3^, Houqing Chen^1^, Changging Jiang^1^, Rui Guo^4,*^, Tao Zhu^5,*^, Shuo Tang^6, *^

^1^ Department of Sport Medicine, Huazhong University of Science and Technology Union Shenzhen Hospital (Nanshan Hospital), Shenzhen, 518000, China

^2^ Department of spine surgery, Huazhong University of Science and Technology Union Shenzhen Hospital (Nanshan Hospital), Shenzhen, 518000, China

^3^ Department of Laboratory Medicine, Huazhong University of Science and Technology Union Shenzhen Hospital (Nanshan Hospital), Shenzhen, 518000, China

^4^ Key Laboratory of Biomaterials of Guangdong Higher Education Institutes, Department of Biomedical Engineering, Jinan University, Guangzhou 510632, China

^5^ Department of Respiratory and Critical Care Medicine, and Preclinical Research Center, Suining Central Hospital, Sichuan, 629000, China

^6^ Department of Orthopaedics, The Eighth Affiliated Hospital, Sun Yat-sen University, Shenzhen, 518000, China

# The authors contributed equally to this work.

*Correspondence: Shuo Tang: tangsh35@[mail.sysu.edu.cn](https://mail.sysu.edu.cn/); Tao Zhu: zhutao063020@163.com; Rui Guo: guorui@jnu.edu.cn;





**Figure S1.** Average pore size of hydrogel.





**Figure S2.** Standard curve of A-CXCL9.





**Figure S3. In vitro release behavior of A-CXCL9.**
